# Luminal androgen receptor subtype and tumor-infiltrating lymphocytes groups based on triple-negative breast cancer molecular subclassification

**DOI:** 10.1038/s41598-024-61640-z

**Published:** 2024-05-17

**Authors:** Miseon Lee, Tae-Kyung Yoo, Byung Joo Chae, Ahwon Lee, Yoon Jin Cha, Jieun Lee, Sung Gwe Ahn, Jun Kang

**Affiliations:** 1 Department of Hospital Pathology, Seoul St. Mary’s Hospital, College of Medicine, The Catholic University of Korea, Seoul, Republic of Korea; 2 Division of Breast Surgery, Department of Surgery, Asan Medical Center, University of Ulsan College of Medicine, Seoul, Republic of Korea; 3 Department of Surgery, Samsung Medical Center, Sungkyunkwan University School of Medicine, Seoul, Republic of Korea; 4 Cancer Research Institute, The Catholic University of Korea, Seoul, Republic of Korea; 5 Department of Pathology, Gangnam Severance Hospital, Yonsei University College of Medicine, Seoul, Republic of Korea; 6 Institute of Breast Cancer Precision Medicine, Yonsei University College of Medicine, Seoul, Republic of Korea; 7 Division of Medical Oncology, Department of Internal Medicine, Seoul St. Mary’s Hospital, The Catholic University of Korea, Seoul, Republic of Korea; 8 Department of Surgery, Gangnam Severance Hospital, Yonsei University College of Medicine, Seoul, Republic of Korea; 9 Institute for Breast Cancer Precision Medicine, Yonsei University College of Medicine, Seoul, Republic of Korea

**Keywords:** Triple-negative breast cancer, Subtype classification, Poly (ADP-ribose) polymerase inhibitors, Homologous recombination deficiency, Tumor-infiltrating lymphocytes, Luminal androgen receptor, Breast cancer, Cancer genomics

## Abstract

In our previous study, we developed a triple-negative breast cancer (TNBC) subtype classification that correlated with the TNBC molecular subclassification. In this study, we aimed to evaluate the predictor variables of this subtype classification on the whole slide and to validate the model’s performance by using an external test set. We explored the characteristics of this subtype classification and investigated genomic alterations, including genomic scar signature scores. First, TNBC was classified into the luminal androgen receptor (LAR) and non-luminal androgen receptor (non-LAR) subtypes based on the AR Allred score (≥ 6 and < 6, respectively). Then, the non-LAR subtype was further classified into the lymphocyte-predominant (LP), lymphocyte-intermediate (LI), and lymphocyte-depleted (LD) groups based on stromal tumor-infiltrating lymphocytes (TILs) (< 20%, > 20% but < 60%, and ≥ 60%, respectively). This classification showed fair agreement with the molecular classification in the test set. The LAR subtype was characterized by a high rate of *PIK3CA* mutation, *CD274* (encodes PD-L1) and *PDCD1LG2* (encodes PD-L2) deletion, and a low homologous recombination deficiency (HRD) score. The non-LAR LD TIL group was characterized by a high frequency of *NOTCH2* and *MYC* amplification and a high HRD score.

## Introduction

Breast cancers that do not express the estrogen receptor (ER) and the progesterone receptor (PR) and that do not overexpress human epidermal growth factor receptor 2 (HER2) are collectively grouped as triple-negative breast cancer (TNBC). This subtype accounts for 15–20% of primary breast cancers^1^ and exhibits more aggressive clinical behavior than other breast cancer subtypes^2,3^. Despite being categorized as a single disease, TNBC is highly heterogeneous^4^.

Researchers at Vanderbilt University, analyzed the gene expression profile of 587 TNBC cases and classified TNBC into six subtypes (the TNBCtype-6 classification)^5^. Basal-like 1 (BL1) is enriched for cell cycle and DNA damage response genes. Basal-like 2 (BL2) is characterized by enrichment in growth factor signaling. Immunomodulatory (IM) is defined by high expression of immune-related pathways. Mesenchymal (M) is characterized by genes related to mesenchymal differentiation and proliferation. Mesenchymal stem–like (MSL) has mesenchymal features but low levels of proliferation. Luminal androgen receptor (LAR) is defined by the activation of hormone-related pathways, specifically through the androgen receptor (AR). Later, the same group refined the molecular subclassification into four subtypes (BL1, BL2, M, and LAR; the TNBCtype-4 classification). This refinement was based on the observation of the significant influence of tumor-infiltrating lymphocytes (TILs) in the IM subtype and stromal cells in the MSL subtype^6^.

In our previous study, we developed a TNBC subtype classification that correlated with the Vanderbilt TNBCtype gene expression molecular subclassification, making TNBC subtyping more widely applicable^7^. We analyzed the gene expression profile of 145 TNBC cases and subtyped them into the re-classified LAR, IM, BL1, and M Vanderbilt TNBC subtypes. We used a classification and regression tree (CART) prediction model for this subtype classification. The predictor variables adopted for the subtype classification in the model were the TIL score and immunohistochemistry (IHC) of the AR and p16, which are commonly used in pathology laboratories. The concordance between this subtype classification and the Vanderbilt subtypes was 0.71. However, we conducted this previous study on a tissue microarray (TMA) for IHC and TIL scores, and it lacked independent external validation. A TMA includes small tissue cores from various regions of the tumor, but it only provides a limited representation of the tumor. By contrast, whole-slide analysis allows for a comprehensive evaluation of the entire tumor and encompasses its full heterogeneity. This approach provides an accurate representation of tumor biology and helps to identify crucial molecular features for subtype classification and characterization. Studies without external validation may encounter several problems, including a lack of generalizability to different datasets, a risk of overestimation of the results, and an inability to assess model performance accurately. Therefore, our proposed TNBC subtype classification requires additional assessment using whole-slide analysis for IHC and external validation to ensure its robustness.

The homologous recombination DNA repair (HRR) pathway is involved in the repair of DNA double-strand breaks^8^. The genomic scar signature score is associated with *BRCA1* and *BRCA2* germline or somatic mutations, *BRCA1* and *RAD51C* promoter methylation, and *PALAB2* germline mutation in TNBC^9,10^. In addition to germline *BRCA1* and *BRCA2* mutations, which affect a key gene in the HRR pathway^11^, somatic or epigenetic *BRCA1* and *BRCA2* alterations, defects in other HRR-modulating genes, and high genomic scar signature scores are emerging as potential predictive markers showing sensitivity to poly (ADP-ribose) polymerase (PARP) inhibitors^12^. The BL1 and BL2 subtypes of the Vanderbilt TNBCtype classification display enrichment of genes associated with cell proliferation and the DNA damage response. In almost all cell lines with mutations in *BRCA1* and *BRCA2*, gene expression is correlated with the basal-like subtype^5^, suggesting that DNA repair–targeting agents are a suitable treatment.

**Figure 1:**
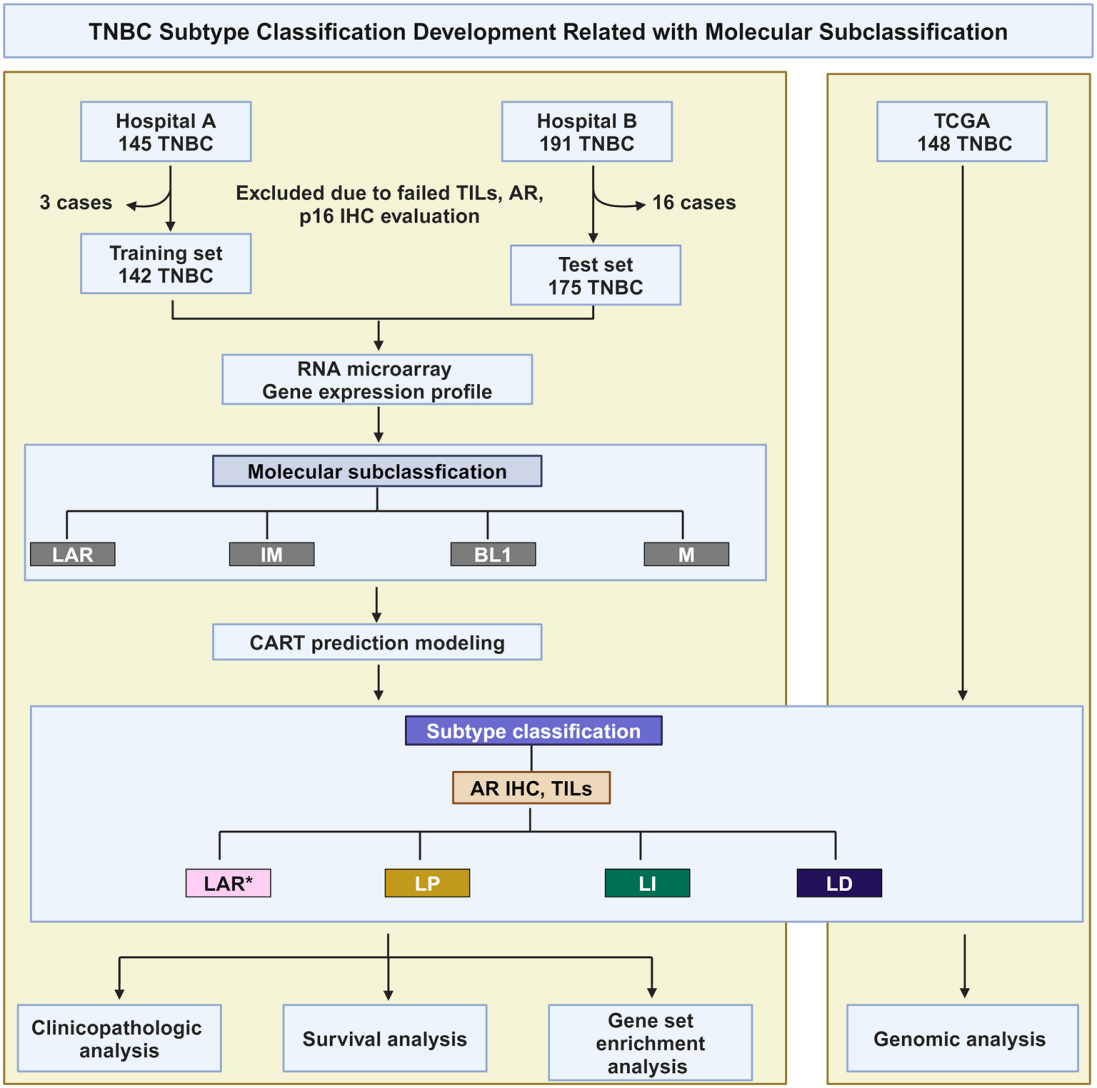
Study design. TNBC, triple-negative breast cancer; TILs, tumor-infiltrating lymphocytes; AR, androgen receptor; IHC, immunohistochemistry; TCGA, The Cancer Genome Atlas; LAR, luminal androgen receptor; IM, immunomodulatory; BL1, basal-like 1; M, mesenchymal; LP, lymphocyte-predominant; LI, lymphocyte-intermediate; LD, lymphocyte-depleted. * Instead of the AR expression results in the TCGA dataset, the Vanderbilt classification based on gene expression was used for the LAR subtypes.

In this study, we evaluated the predictor variables of our previous TNBC subtype classification, p16 and AR expression and TIL scores, on whole slides, which are used in pathology laboratories, rather than TMAs to capture the overall tumor biology and to improve subtype classification. In addition, we sought to validate the model’s performance by using an external test set to assess its robustness. We then explored the characteristics of this subtype classification and investigated genomic alterations, including genomic scar signature scores. The study design is shown in Fig. 1.

## Results

### Clinical characteristics and gene expression-based TNBC molecular subclassification of the training and test sets

The training set had a significantly higher frequency of metaplastic carcinoma and a significantly higher histological grade than the test set. Age, tumor size, and the American Joint Committee on Cancer (AJCC) pathologic stage were not significantly different between the training and test sets (Supplementary Table S1).

According to the TNBC molecular subclassification, 142 TNBC cases in the training set and 175 TNBC cases in the test set were assigned to one of the LAR, IM, BL1, or M subtypes. In the training set, there were 21 cases (14.8%) of the LAR subtype, 31 cases (21.8%) of the IM subtype, 25 cases (17.6%) of the BL1 subtype, and 38 cases (26.8%) of the M subtype. In the test set, there were 24 cases (13.7%) of the LAR subtype, 31 cases (17.7%) of the IM subtype, 37 cases (21.1%) of the BL1 subtype, and 42 cases (24.0%) of the M subtype. There were 27 unclassified cases (19.9%) in the training set and 41 unclassified cases (23.4%) in the test set; we excluded these cases. There was no significant difference in the TNBC molecular subclassification between the two sets (*p* = 0.700) (Supplementary Table S1).

### Subtype classification of TNBC into LAR subtype and TIL groups

**Figure 2:**
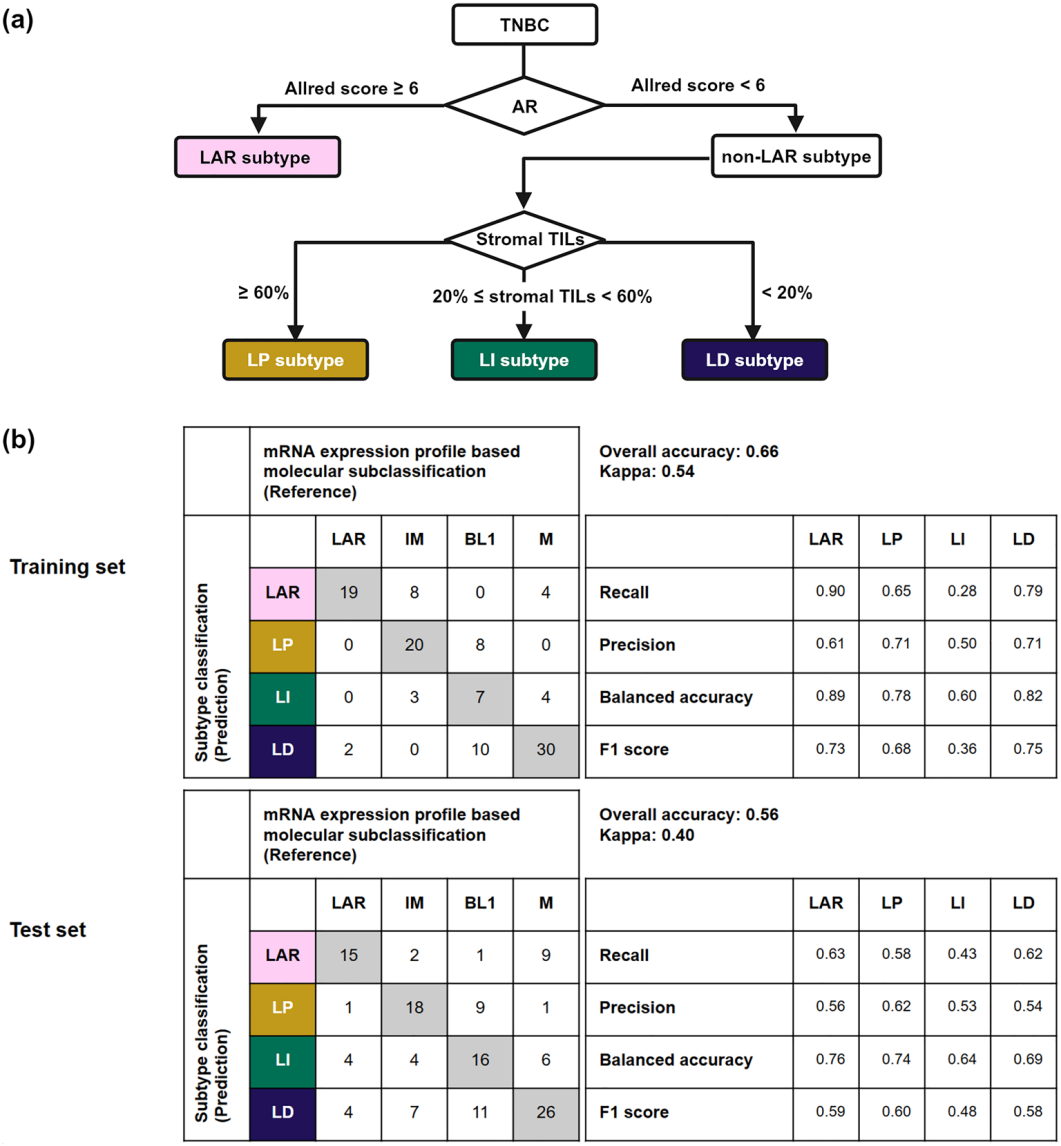
Triple-negative breast cancer (TNBC) subtype classification and performance metrics. (**a**) Decision tree for classifying the TNBC subtypes based on the AR Allred score and percentage of stromal TILs, dividing cases into the LAR, LP, LI, and LD subtypes. (**b**) Performance of the classification and regression tree prediction model in the training and test sets, evaluated with the accuracy metrics recall, precision, balanced accuracy, the F1 score, overall accuracy, and the kappa statistic. AR, androgen receptor; LAR, luminal androgen receptor; TILs, tumor-infiltrating lymphocytes; LP, lymphocyte-predominant; LI, lymphocyte-intermediate; LD, lymphocyte-depleted.

The CART prediction model created a subtype classification that corresponds to the TNBC molecular subclassification. We selected the AR Allred score and the TIL score as markers for this subtype classification, but we excluded the p16 staining pattern. The CART prediction model selected a cut-off of 6 points for the AR Allred score and 20% and 60% for the TIL score. Based on the prediction of the CART model, this subtype classification first classified TNBC into two subtypes according to the AR Allred score: the LAR subtype for a score ≥ 6, and the non-LAR subtype for a score < 6. Next, the non-LAR subtype was further subdivided into three TIL groups with the TIL score cut-offs of 20% and 60%: the lymphocyte-predominant (LP), lymphocyte-intermediate (LI), and lymphocyte-depleted (LD) groups. The subtype classification was as follows: LAR subtype, AR Allred score ≥ 6; non-LAR subtype LP group (abbreviated as the LP group), AR Allred score < 6 and TIL score ≥ 60%; non-LAR subtype LI group (abbreviated as the LI group), AR Allred score < 6 and 20% ≤ TIL score < 60%; non-LAR subtype LD group (abbreviated as the LD group), AR Allred score < 6 and TIL score < 20% (Fig. 2). The LAR subtype corresponded to the LAR molecular subtype. Among the non-LAR subtypes, the LP group corresponded to the IM molecular subtype, the LI group to the BL1 molecular subtype, and the LD group to the M molecular subtype.

### F1 score and concordance between the subtype classification and the TNBC molecular subclassification

The overall concordance between the subtype classification and the TNBC molecular subclassification was 0.66 (95% confidence interval [CI] 0.57–0.75; κ = 0.54) in the training set and 0.56 (95% CI 0.47–0.65; κ = 0.40) in the test set. In both sets, the LP group showed the highest precision (also called the positive predictive value), while the LI group showed the lowest precision among the four subtypes. The LAR subtype had the highest recall (referred to as sensitivity), while the LI group had the lowest, similarly to its precision. The F1 score of the LI group was the lowest, reflecting its precision and recall values (Fig. 2b).

### The differences in clinicopathologic characteristics among the LAR subtype and the TIL groups

Among 317 cases, including those in the training and test sets, the LAR subtype comprised 71 cases (22.3%), the LP group included 73 cases (23.0%), the LI group included 59 cases (18.6%), and the LD group included 114 cases (36.0%). The age at diagnosis, histologic type, and tumor size showed significant differences among the LAR subtype and the TIL groups. The LAR subtype was associated with the oldest age. Although the number was small, all four cases of invasive lobular carcinoma had the LAR subtype, and all four cases of salivary gland–like carcinoma were in the LD group. Carcinoma with medullary features was the most frequent in the LP group, and metaplastic carcinoma was the most common in the LD group. The LAR subtype showed a lower histologic grade than the non-LAR subtype. The LP group had the smallest tumor size. There was no significant difference in the AJCC pathologic stage among the subtypes (Table 1).

**Table 1:** Clinical characteristics and the subtype classification in the training set and test set.

	LAR (N = 71)	LP (N = 73)	LI (N = 59)	LD (N = 114)	*p*-value
Age, years (mean (SD))	59.2 (31.8)	48.7 (12.1)	51.1 (11.6)	52.5 (11.3)	0.005
Histologic diagnosis, N (%)					0.004
Invasive breast carcinoma, NST	55 (77.5)	55 (75.3)	47 (79.7)	86 (75.4)	
Carcinoma with medullary features	4 (5.6)	12 (16.4)	6 (10.2)	5 (4.4)	
Metaplastic carcinoma	4 (5.6)	3 (4.1)	2 (3.4)	14 (12.3)	
Carcinoma with apocrine differentiation	3 (4.2)	2 (2.7)	2 (3.4)	2 (1.8)	
Invasive lobular carcinoma	4 (5.6)	0 (0.0)	0 (0.0)	0 (0.0)	
Salivary gland-like carcinoma	0 (0.0)	0 (0.0)	0 (0.0)	4 (3.5)	
Other	1 (1.4)	1 (1.4)	2 (3.4)	3 (2.6)	
Histologic grade, N (%)					< 0.001
1	7 (10.1)	0 (0.0)	1 (1.8)	3 (2.7)	
2	25 (36.2)	8 (11.1)	9 (15.8)	16 (14.3)	
3	37 (53.6)	64 (88.9)	47 (82.5)	93 (83.0)	
Tumor size, cm (mean (SD))	2.4 (1.2)	2.1 (0.8)	2.7 (1.4)	2.7 (1.7)	0.028
AJCC pathologic stage, N (%)					0.280
1	26 (36.6)	31 (42.5)	15 (25.4)	42 (36.8)	
2	38 (53.5)	37 (50.7)	37 (62.7)	62 (54.4)	
3	7 (9.9)	5 (6.8)	7 (11.9)	7 (6.1)	
4	0 (0.0)	0 (0.0)	0 (0.0)	3 (2.6)	
TNBC molecular subclassification, N (%)					< 0.001
LAR	34 (47.9)	1 (1.4)	4 (6.8)	6 (5.3)	
IM	10 (14.1)	38 (52.1)	7 (11.9)	7 (6.1)	
BL1	1 (1.4)	17 (23.3)	23 (39.0)	21 (18.4)	
M	13 (18.3)	1 (1.4)	10 (16.9)	56 (49.1)	
UNC	13 (18.3)	16 (21.9)	15 (25.4)	24 (21.1)	

LAR, Luminal androgen receptor; LP, Lymphocyte-predominant; LI, Lymphocyte-intermediate; LD, Lymphocyte-depleted; SD, standard deviation; NST, no special type; AJCC, The American Joint Committee on Cancer; IM, Immunomodulatory; BL1, basal-like 1; M, Mesenchymal; UNC, Unclassified.

### Log-rank test and multivariable cox regression analysis in the LAR subtype and the TIL groups

**Figure 3:**
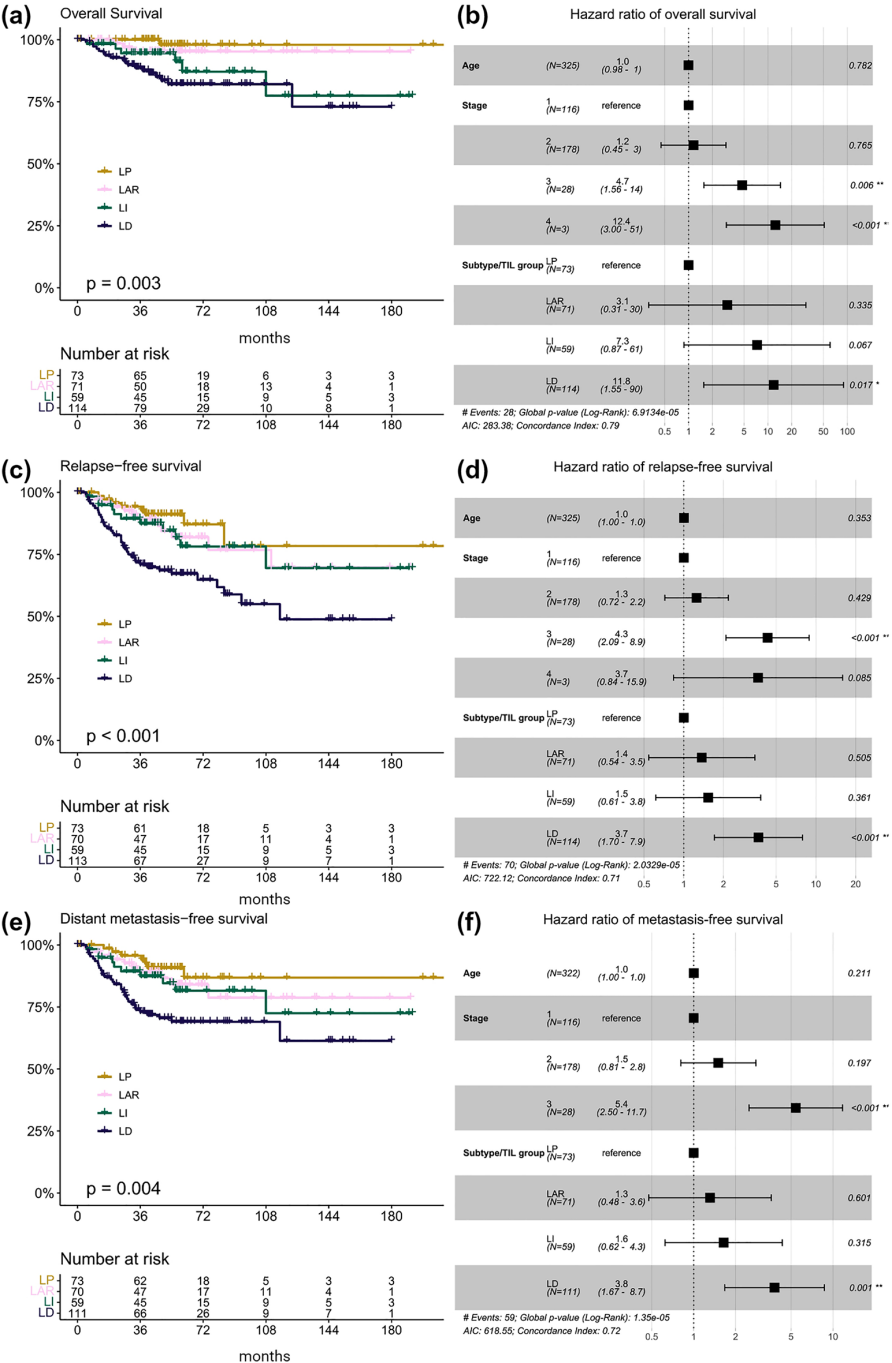
Survival analysis. Kaplan–Meier curves and the forest plots for multivariable Cox proportional hazards regression for overall survival (**a**, **b**), relapse-free survival (**c**, **d**), and distant metastasis-free survival (**e**, **f**) according to the triple-negative breast cancer (TNBC) subtype classification. LAR, luminal androgen receptor; TILs, tumor-infiltrating lymphocytes; LP, lymphocyte-predominant; LI, lymphocyte-intermediate; LD, lymphocyte-depleted.

The log-rank test showed significant differences in survival outcomes among the subtypes for overall survival (OS) (*p* = 0.003), relapse-free survival (RFS) (*p* < 0.001), and distant metastasis–free survival (DFS) (*p* = 0.004) (Fig. 2). In the multivariable survival analysis, the LD group was an independent prognostic factor for poor OS, RFS, and DFS. The LD group had significantly worse OS than the LP group (hazard ratio [HR] 11.8, 95% CI 1.55–89.7, *p* = 0.017). The risk of recurrence was significantly higher in the LD group than in the LP group (HR 3.68, 95% CI 1.70–7.93, *p* < 0.001). The risk of metastasis was significantly higher in the LD group than in the LP group (HR 3.81, 95% CI 1.67–8.67, *p* = 0.001) (Fig. 3). In separate analyses of the training and test sets, the LD subtype was associated with significantly worse prognosis for OS, RFS, and DFS in the training set. This association was not found to be statistically significant in the test set (Supplementary Fig. 1).

### Hallmark signatures of the LAR subtype and the TIL groups

**Figure 4:**
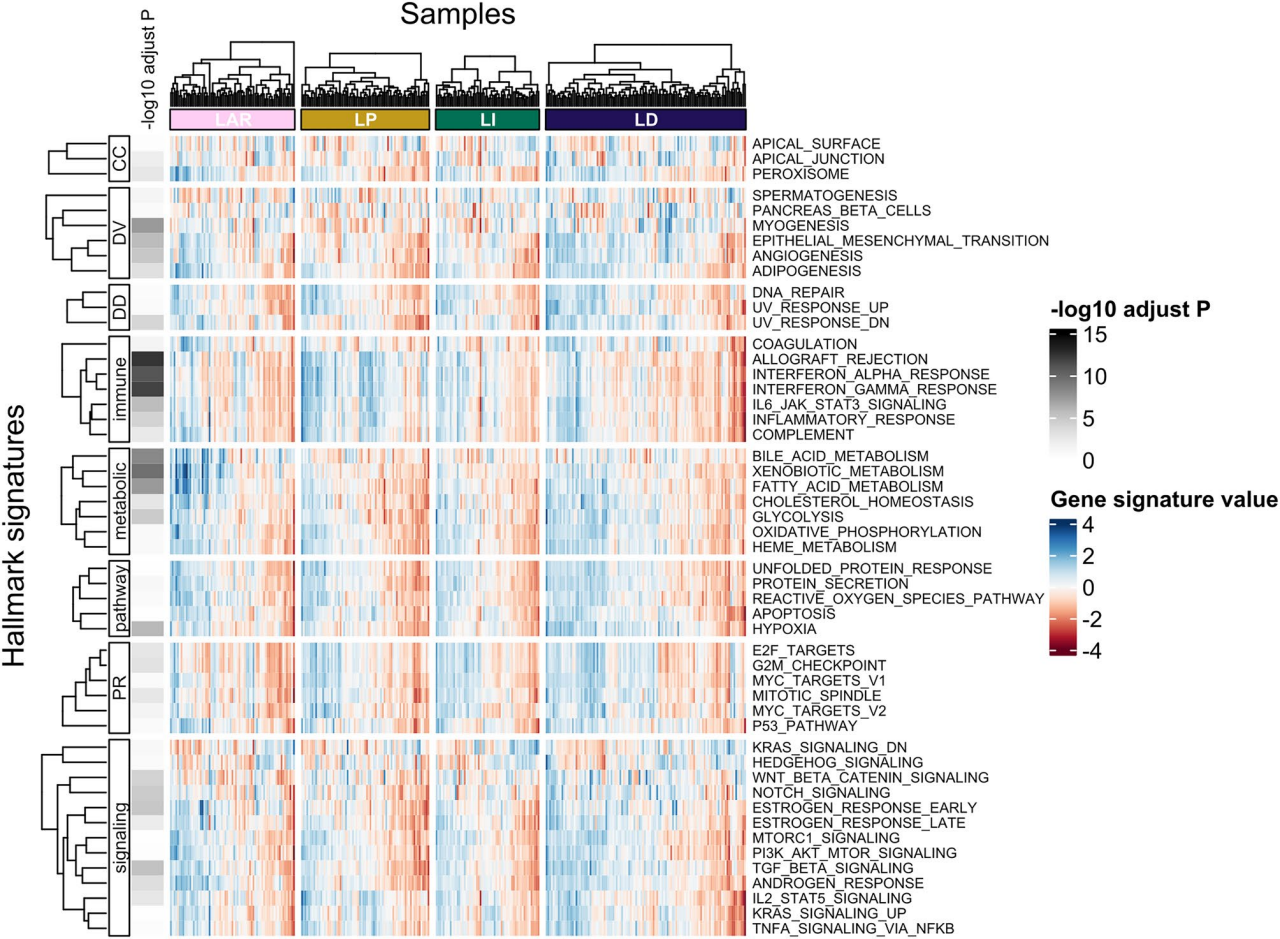
Hallmark signatures and differences according to the triple-negative breast cancer (TNBC) subtype classification. Each cell in the heatmap represents the gene signature value. Each column represents samples grouped by subtype classification. Each row represents a hallmark signature grouped by category. The left column with annotation represents the minus logarithm (base 10) of the adjusted *p*-values. This heatmap is generated using the tidyHeatmap R package (version 1.8.1). LAR, luminal androgen receptor; LP, lymphocyte-predominant; LI, lymphocyte-intermediate; LD, lymphocyte-depleted; CC, cellular component; DD, DNA damage; DV, development; PR, proliferation.

**Figure 5:**
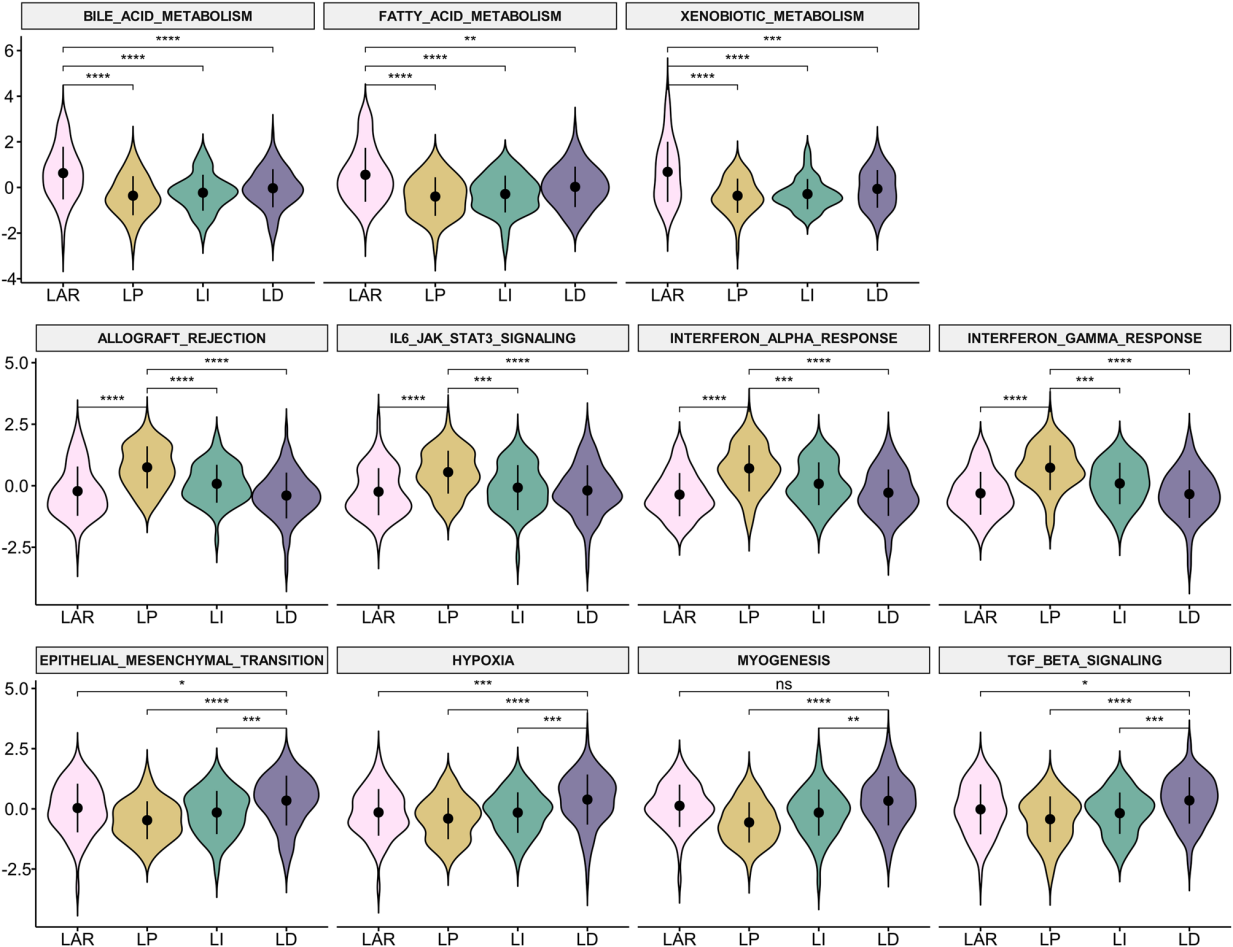
Violin plots of significantly different hallmark signatures according to the triple-negative breast cancer (TNBC) subtype classification. Post hoc analysis of hallmark signatures with an adjusted *p* < 1 × 10^−5^ was performed. The first row of signatures is related to metabolism; the second to immunity; and the third to epithelial-mesenchymal transition, hypoxia, myogenesis, and transforming growth factor-β. LAR, luminal androgen receptor; LP, lymphocyte-predominant; LI, lymphocyte-intermediate; LD, lymphocyte-depleted; ns, *p* > 0.05; *p ≤ 0.05; **p ≤ 0.01; ***p ≤ 0.001; ****p = 0.0001.

Of the 50 hallmark signatures, 31 were significantly different among the LAR subtype and the TIL groups. Eleven hallmark signatures had an adjusted *p* < 1 × 10^−5^. The LAR subtype showed a significantly higher gene set enrichment analysis (GSEA) score than the TIL groups in metabolism hallmark signatures, including bile acid metabolism, fatty acid metabolism, and xenobiotic metabolism. The LP group showed a significantly higher GSEA score than the other TIL groups and the LAR subtype in immune hallmark signatures, including allograft rejection, IL6 JAK STAT3 signaling, the interferon-alpha response, and the interferon-gamma response. The LD group showed a significantly higher GSEA score for epithelial–mesenchymal transition, hypoxia, myogenesis, and transforming growth factor (TGF)-beta. The GSEA scores are presented graphically in Fig. 4. The 11 hallmark signatures with an adjusted *p* < 1 × 10^−5^ are summarized in Fig. 5.

### Genomic alterations in the LAR subtype and the TIL groups using The Cancer Genome Atlas (TCGA) dataset

From the TNBC molecular subclassification evaluated by Bareche et al.^13^ and whole hematoxylin and eosin (H&E) slide images from TCGA, 148 TNBC cases were available for subtype classification into the LAR subtype and the TIL groups in the firehose dataset, and 76 cases were available in the TCGA invasive breast cancer/Nature 2012 dataset. In the firehose dataset, there were 26 cases (16.1%) in the LAR subtype, 21 cases (13.0%) in the LP group, 34 cases (21.1%) in the LI group, and 80 cases (49.7%) in the LD group. In the TCGA invasive breast cancer/Nature 2012 dataset, there were 9 cases (11.8%) in the LAR subtype, 14 cases (18.4%) in the LP group, 15 cases (19.7%) in the LI group, and 38 cases (50.5%) in the LD group.

**Figure 6:**
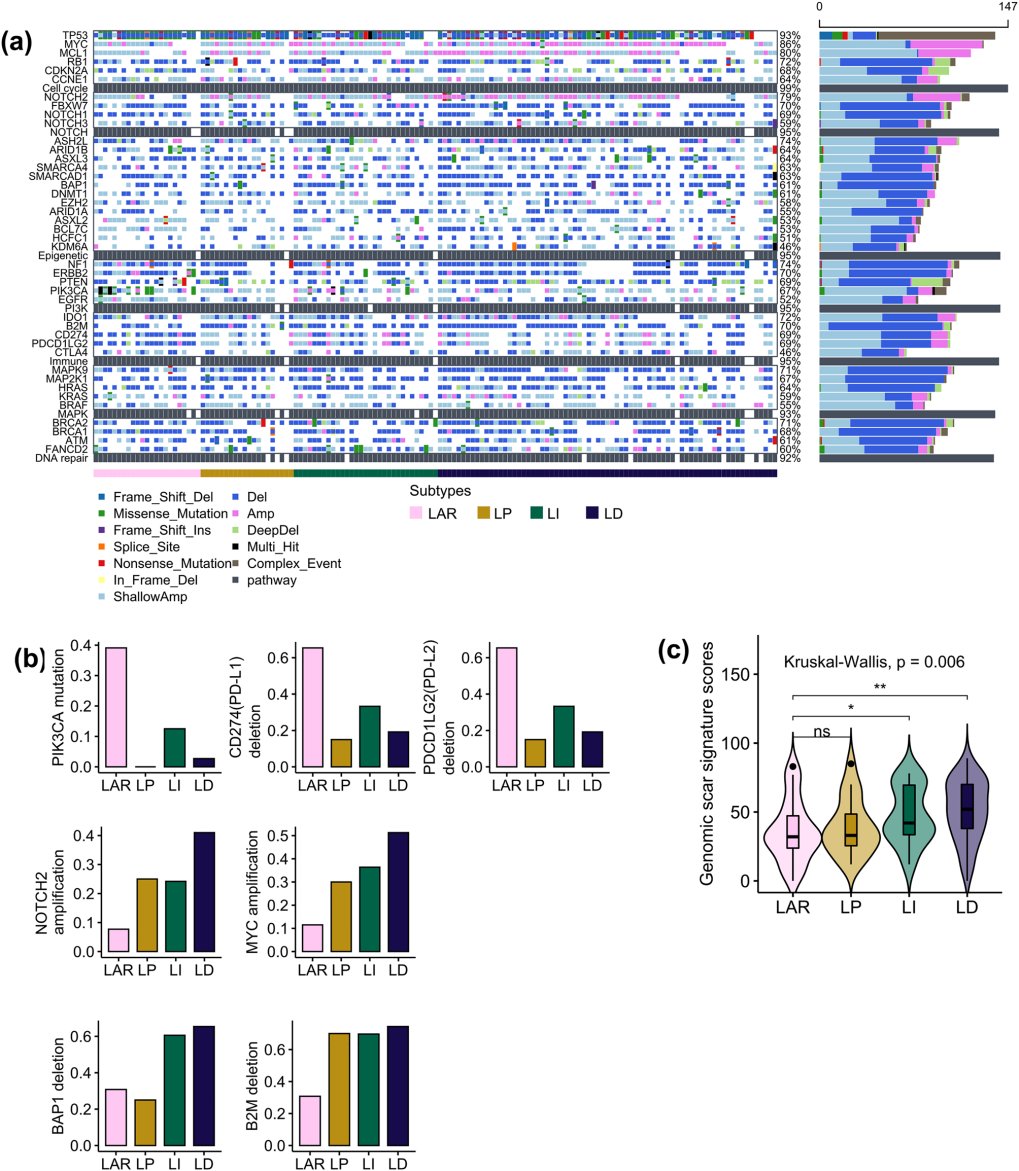
Genomic analysis of the triple-negative breast cancer (TNBC) subtype classification in The Cancer Genome Atlas (TCGA) dataset. (**a**) Oncoplot visualizing the frequency of molecular alterations across samples, categorized by the TNBC subtype (LAR, LP, LI, LD). Alterations are depicted by color-coded mutation types. The bar plot on the right indicates the total number of alterations per sample. This oncoplot is generated using the maftools R package (version 2.16.0). (**b**) Bar plots of significantly different genomic alterations. (**c**) Violin plot for the genomic scar signature. The difference in genomic scar signature scores was statistically evaluated using the Kruskal–Wallis test followed by the post hoc Wilcoxon signed-rank test. LAR, luminal androgen receptor; LP, lymphocyte-predominant; LI, lymphocyte-intermediate; LD, lymphocyte-depleted.

The LAR subtype was characterized by a high *PIK3CA* mutation rate, high *CD274* (encodes PD-L1) and *PDCD1LG2* (encodes PD-L2) deletion, and a low genomic scar signature score. The LD group was characterized by a high *NOTCH2* and *MYC* amplification rate and a high genomic scar signature score. The non-LAR subtypes were characterized by a low *CD274* and *PDCD1LG2* deletion rate and a high *B2M* deletion rate compared with the LAR subtype. The frequency of *BRCA1* and *BRCA2* germline and somatic mutations did not show significant differences among the LAR subtype and the TIL groups. The genomic scar signature score was significantly higher in the LI and LD groups than in the LAR subtype (Fig. 6).

## Discussion

In this study, we attempted to classify TNBC into subtypes that correlate with the TNBC molecular subclassification by using whole-slide images to evaluate TIL scores and IHC of p16 and AR, which were selected as predictor variables from the previous subtype classification. The CART prediction model selected the AR Allred score (cut-off: 6) and the TIL score (cut-off: 20% and 60%) as the predictor variables, but not the p16 staining pattern. TNBC was categorized into LAR and non-LAR subtypes, and the non-LAR subtype was further divided into the TIL groups. The overall accuracy of subtype classification into the TNBC molecular subclassification was 0.66 (κ = 0.54) in the training set and 0.56 (κ = 0.40) in the test set. Although this subtype classification showed low agreement with the TNBC molecular subclassification, each of the four subtypes in this classification displayed specific characteristics. The distinct clinicopathologic and genomic features of the proposed TNBC subtype classification is summarized in Table 2. Our proposed AR Allred score and TIL score cut-offs are supported by evidence from clinical and genetic analyses, which have been used arbitrarily in previous studies. In addition, our findings suggest that only AR IHC and the TIL scores could be used to subtype TNBC, indicating the potential for a clinically feasible TNBC stratification approach.

**Table 2:** Summary of the distinct clinicopathologic and genomic characteristics of the proposed TNBC subtype classification.

TNBC subtype classification	LAR	non-LAR		
		LP	LI	LD
Definition	AR Allred score ≥ 6	AR Allred score < 6		
		Stromal TILs ≥ 60%	Stromal > 20% TILs < 60%	Stromal TILs < 20%
Molecular subclassification	LAR	IM	BL1	M
Clinicopathologic characteristics	Oldest age Invasive lobular carcinoma Lower histologic grade	Carcinoma with medullary features Smallest tumor size		Salivary gland-like carcinoma Metaplastic carcinoma
Survival	Favorable OS, RFS	Favorable OS, RFS, and DFS		Poor OS, RFS, and DFS
Hallmark signatures	Metabolism hallmark signatures, including acid metabolism, fatty acid metabolism, and xenobiotic metabolism	Immune hallmark signatures, including allograft rejection, IL6 JAK STAT3 signaling, the interferon-alpha response, and the interferon-gamma response		Epithelial-mesenchymal transition, hypoxia, myogenesis, and transforming growth factor (TGF)-beta
Genomic alterations	High *PIK3CA mutation* High *CD274* (*PD-L*1) and *PDCD1LG2* (*PD-L*2) deletion Low HRD score	High *B2M* deletion		
				High *NOTCH2 and MYC* amplification High HRD score

LAR, Luminal androgen receptor; LP, Lymphocyte-predominant; LI, Lymphocyte-intermediate; LD, Lymphocyte-depleted; AR, Androgen receptor; TILs, tumor-infiltrating lymphocytes; IM, Immunomodulatory; BL1, basal-like 1; M, Mesenchymal; overall survival (OS), relapse-free survival (RFS), and distant metastasis-free survival (DFS).

AR is expressed in approximately 10–43% of TNBC cases, depending on the definition of AR positivity used. Some studies used a threshold of more than 10% of tumor cells showing AR staining^14^, while others used an Allred score of ≥ 3, similar to the criteria used for estrogen receptor (ER) and progesterone receptor (PR) positivity^15^. The H-score method has also been used to determine positive AR expression^16^. These variations in the definition of positive AR expression may lead to confusion, and the prevalence rates of AR-positive TNBC cases may vary among studies due to the use of different criteria. Positive criteria for ER and PR expression have been crucial to classify breast cancer and to guide hormonal therapy decisions, and substantial research and effort have been dedicated to establishing the current criteria. Likewise, the establishment of the criteria for defining positive AR expression is also necessary for the accurate classification of TNBC. In this study, based on TNBC molecular subclassification, we proposed a criterion for AR expression that allowed the division of TNBC cases into the LAR and non-LAR subtypes. The LAR subtype showed distinct characteristics, such as diagnosis at an older age and a lower histologic grade compared with the non-LAR subtype. This subtype displayed hallmark signatures associated with metabolism, a high rate of *PIK3CA* mutation and *CD274* and *PDCD1LG2* deletion, and a low genomic scar signature score. Similarly to our study, previous research has shown that the LAR subtype is associated with a high frequency of older age and invasive lobular carcinoma^17^, and enriched gene signatures are associated with hormone-related gene signaling pathways (including steroid hormone, porphyrin metabolism, and androgen/estrogen metabolism) and a high frequency of *PIK3CA* mutations^5^. Studies have reported that patients with AR-positive TNBC showed better DFS^18^ and a more favorable response to neoadjuvant chemotherapy^19^. We observed that the LAR subtype also showed a favorable prognosis in terms of OS, RFS, and DFS. Hence, it is possible that an Allred score of 6, as determined by molecular subclassification, could serve as a potential cut-off for the LAR subtype.

Recently, AR-negative TNBC, which accounts for approximately 67%–90% of TNBC cases, has been considered a separate molecular subtype from AR-positive TNBC and is referred to as quadruple-negative breast cancer (QNBC)^20,21^. QNBC does not benefit from AR antagonists. Studies suggesting a worse prognosis for QNBC^22^ compared with AR-positive TNBC indicate the need to understand the biological basis and to explore alternative therapeutic strategies for QNBC. Recent research has uncovered fatty acyl-CoA synthetase 4, S-phase kinase associated protein 2, EGFR, and CD151 as potential candidates^20^. As mentioned above, the variability in the definition of AR positivity complicates the study of QNBC. The non-LAR subtype proposed in our study, based on gene expression results and with evidence of distinctive features, could be used as a surrogate for QNBC. The poorer prognosis of the non-LAR subtype compared with the LAR subtype is consistent with QNBC. The non-LAR subtype may help to define QNBC in future studies.

Studies have reported that high TILs can have predictive value for the treatment efficacy of immune checkpoint inhibitors^23^ or neoadjuvant chemotherapy^24^, and are related to a better response, a pathologic complete response (pCR), and survival^25,26^. According to the 2014 International TILs Working Group^27^, the level of TILs is assessed using 10% increments. However, it appears that stratification of TIL levels is necessary in practice. Categorizing TILs into groups, such as low, intermediate, and high, improves the interpretability of the results, supports clinical decision making, and allows for better comparison and standardization across studies. This approach can facilitate clinical translation, making it easier for researchers and clinicians to analyze and communicate the significance of TILs in different contexts. In this study, we divided TILs into three groups based on molecular subclassification, each with distinct characteristics. The LP group had a high frequency of carcinoma with medullary features and the smallest tumor size, immune hallmark signatures, and good OS. The LD group showed a high frequency of metaplastic carcinoma and salivary gland-like carcinoma; significantly poorer OS, RFS, and DFS; the mutational signature of epithelial–mesenchymal transition, hypoxia, myogenesis, and TGF-beta; a high rate of *NOTCH2* and *MYC* amplification; and a high genomic scar signature score. The identification of distinct characteristics and clinical outcomes associated with each TIL group suggests that subdivision into TIL groups with 20% and 60% cut-offs for the TIL score may be valid.

In our genomic analysis using the TCGA dataset, the genomic scar signature score was higher in the LI and LD groups than in the LAR subtype or the LP group. Our subtype classification suggests that high genomic scar signature scores are related to the non-LAR subtype and lower TILs. This result suggests that our subtype classification could potentially be applied to stratify patients with TNBC in clinical trials for treatment with PARP inhibitors.

A recent study^28^, which classified TNBC molecular subtypes by using gene expression microarrays, reported that the LAR molecular subtype showed favorable OS and DFS. The IM molecular subtype demonstrated relatively better DFS, while the M and BL2 molecular subtypes exhibited worse OS and DFS. Regarding the 3-year recurrence rate, the LAR molecular subtype showed a significantly better prognosis than the M and BL2 molecular subtypes. The results of our survival analysis were in close agreement to that study. In our study, the subtypes exhibited significantly different survival rates. The LAR subtype showed favorable OS, RFS, and DFS. For the non-LAR subtype, the LP group showed significantly better OS, RFS, and DFS compared with the LD group. These results support the idea that our subtype classification can serve as a prognostic marker. However, the lack of statistical significance in the test set suggests the need for further investigation. It highlights the need for additional studies with larger sample sizes to robustly validate the prognostic utility of our subtype classification.

This study has some limitations. There was relatively low agreement between our proposed TNBC subtype classification (LAR, LP, LI, and LD) and the re-classified Vanderbilt TNBCtype-4 classification (LAR, IM, BL1, and M), with an overall accuracy of 0.56 in the test set. The LI group contributed the most to lowering the overall accuracy among the four subtypes. The precision, recall, and F1 score were the lowest for BL1 molecular subtype classification in the LI group. The LI group did not show any distinct signature in our gene expression profile analysis, unlike the BL1 molecular subtype, which showed enrichment of genes involved in the cell cycle and DNA damage response (ATR/BRCA) pathway^29,30^. The LI group is probably different from the BL1 molecular subtype, and further research on this group is required.

TNBC clinically defined by ER, PR, and HER2 expression based on IHC differs from TNBC within the TCGA dataset defined by gene expression profiles. In addition, there were no AR expression results or AR scores based on IHC, so we could not divide the TNBC cases of the TCGA datasets into LAR and non-LAR subtypes by AR Allred score. So, we just defined the LAR molecular subtype of the Vanderbilt classification as the LAR subtype. To overcome this problem, we are conducting further research to characterize the genomic alterations of subtypes in clinically defined TNBC.

Several studies have attempted to classify TNBC subtypes using different IHC marker panels^31–33^. Although these studies have shown that TNBC classification was possible only with the IHC panel, they lacked comparisons with gene expression profiles. A study attempted IHC-based classification with a substantial agreement with messenger RNA (mRNA) expression–based FUSCC molecular subtyping^34^. This classification used AR, CD8, FOXC1, and DCL1 IHC to classify TNBC into the LAR, IM, BLIS, MES, and unclassified groups. This IHC-based classification was suggested as an independent prognostic factor for RFS in multivariable survival analysis. Indeed, this classification showed similarity to our subtype classification. However, our subtype classification is simpler, and our study evaluated genomic alterations in addition to gene expression signatures for each subtype.

In conclusion, we have presented a TNBC subtype classification that is easy to apply in pathology laboratories and correlates with molecular subclassification based on mRNA expression. Despite the low agreement with gene expression molecular subclassification, the TNBC subtype classification demonstrates distinct clinical and genomic characteristics. Our findings support a proposed AR Allred score cut-off of 6 for categorizing the LAR and non-LAR subtypes, and TIL score cut-offs of 20% and 60% for categorizing the TIL groups of the non-LAR subtype. These cut-offs appear to be useful for stratifying TNBC.

## Methods

### Dataset collection

The training set included the same 145 TNBC cases from Seoul St. Mary’s Hospital between January 2009 and October 2017 as in the previous study for the original subtype classification^7^. The test set comprised 191 cases of TNBC from Gangnam Severance Hospital from June 1997 to November 2014. After excluding the cases that failed the TIL score evaluation or IHC for AR and p16, the final training and test sets included 142 and 175 cases, respectively. The detailed inclusion and exclusion criteria were described in previous studies^7,35^. The need for informed consent was waived by the institutional review boards of Seoul St. Mary’s Hospital (KC21SISI0597) and Gangnam Severance Hospital (3-2013-0268).

### RNA microarray and gene expression profile

RNA microarray analysis was performed to obtain gene expression data. Whole tissue and the same representative paraffin-fixed formalin-embedded tissue blocks employed for the microarray analysis were used. The Affymetrix Human Gene 2.0 ST Array and the Human Genome U133 Plus 2.0 Array were used in the training and test sets, respectively. The detailed protocols for total RNA isolation, RNA extraction, purification, labeling, hybridization, and the microarray assay were described in previous studies^7,35^. Raw gene expression profiles were normalized by quantile methods, log2 transformed, and centered around the median.

### Gene expression-based TNBC molecular subclassification

Gene expression profiles from the microarray analysis of the training and test sets were uploaded to the TNBCtype website (http://cbc.mc.vanderbilt.edu/tnbc/) to classify TNBC into the Vanderbilt TNBCtype. For each case, correlation scores and corresponding *p*-values were obtained for each the six original subtypes (Vanderbilt TNBCtype: LAR, BL1, BL2, IM, M, and MSL)^5^.

Lehmann et al.^5^ refined the TNBC classification from six to four subtypes (LAR, BL1, BL2, and M) after observing the influence of TILs in the IM subtype and stromal cells in the MSL subtype. Bareche et al.^13^ observed that the BL2 and unstable (UNS) subtypes clustered non-specifically, indicating their lower reproducibility. However, given the importance of the immune response and the potential for immunotherapy in TNBC, it was necessary to maintain the IM subtype. Finally, we selected the LAR, IM, BL1, and M subtypes and named this re-classified Vanderbilt classification as the TNBC molecular subclassification. Then, we then proposed a subtype classification that could predict this TNBC molecular subclassification, as in the previous study. Each case was assigned to one of four subtypes with the highest correlation score and *p* < 0.05. Cases with *p* ≥ 0.05 for all four subtypes represented an unclassified group; we excluded these cases.

### Evaluation of p16 and AR expression and TILs, which were selected as predictor variables in the previous subtype classification

P16 and AR expression and TIL scores, which were predictor variables in the previous subtype classification^7^, were evaluated in one whole representative section of each case from the training and test sets. An automatic IHC staining device (Ventana BenchMark ULTRA; Roche, USA) and antibodies targeting p16 (E6H4; Roche) and AR (SP107; Roche) were used. For p16, staining patterns were classified as negative, weak and mosaic, or diffuse and strong. AR was assessed as an Allred score, following the guidelines of St. Gallen and the American Society of Clinical Oncology/College of American Pathologists for ER and PR statining^36,37^ due to their similarity. The Allred score comprises a proportion score that reflects the percentage of AR-positive cells detected by immunohistochemistry (assigned 0–5 points for 0%, ≤ 1%, 1–10%, 11–33%, 34–66%, and ≥ 67%, respectively) and a nuclear intensity score (assigned 0, 1, 2, and 3 points for negative, weak, intermediate, and strong staining, respectively). Thus, the Allred score ranges from 0 to 8 points. The TIL score was determined by evaluating stromal TILs on the H&E-stained histology slides. The level of stromal TILs was assessed according to the 2014 International TILs Working Group and scored using 10% increments^27^.

### CART prediction modeling

CART modeling was performed to predict the TNBC molecular subclassification using p16 and AR expression and TIL scores as predictor variables. The R package version rpart 4.1.19 was used for the modelling process. The parameters were set to 5 for maximum depth and 0.0001 for complexity. The subtype classification was developed based on the CART prediction model that correlates with the molecular subclassification. The performance of the subtype classification was evaluated in the training and test sets.

### GSEA according to subtype classification

GSEA was performed on 336 cases, both the training and test sets, to investigate the associated pathways for each subtype^38^. The R package IOBR version 0.99.8 was used^39^. The difference in the signature score among the four subtypes was statistically evaluated with analysis of variance (ANOVA), and *p*-value adjustments were made using the false discovery rate (FDR). Post hoc analysis was conducted with Student’s t-test. The significance level was set at an adjusted *p* < 0.05.

### Survival analysis according to subtype classification

The survival analysis was conducted on the entire dataset (n = 336), including the training and test sets, as well as separate analyses for each set. Kaplan–Meier survival curves and the log-rank test were used to compare the OS, RFS, and DFS among the subtypes. The Cox proportional-hazards test was performed for multivariable survival analysis, and the HR and its 95% CI were estimated. The significance level was set at *p* < 0.05.

### Subtype classification of the TCGA dataset and genomic analysis

Genomic alterations of the subtype classification were investigated using genomic data from TCGA, namely the firehose dataset and the TCGA invasive breast cancer/Nature 2012 dataset downloaded from cBioPortal (https://www.cbioportal.org/). The firehose dataset was used to evaluate somatic mutations and copy number alterations. The TCGA invasive breast cancer/Nature 2012 dataset^40^ was used to evaluate *BRCA1* and *BRCA2* germline and somatic mutations.

The TNBC molecular subclassification in TCGA cases was previously evaluated by Bareche et al.^13^ and is available on the journal’s website. Therefore, TNBC cases within the TCGA dataset could be classified with the LAR subtype. The TIL score could be evaluated from the archived H&E-stained digital slide images (https://cancer.digitalslidearchive.org/).

Genomic alterations were analyzed according to subtype classification. Forty-two genes were selected according to a previous study by Lehmann et al.^17^. The frequency of mutations, gene deletions, and amplifications were analyzed according to subtype. Both homozygous and heterozygous deletions were considered gene deletions. Amplification, but not low level gain, was compared. The R package maftools version 2.16.0 was used to summarize and visualize mutations and copy number changes. Fisher's exact test and an FDR correction were applied. The significance level was set at an adjusted *p* < 0.05.

The genomic scar signature scores were estimated by Marquard et al.^41^ and downloaded from the journal’s website. The difference in genomic scar signature scores was statistically evaluated with the Kruskal–Wallis test followed by the post hoc Wilcoxon signed-rank test.

### Ethics approval

The institutional review boards of Seoul St. Mary’s Hospital (KC21SISI0597) and Gangnam Severance hospital (3-2013-0268) approved this study. All procedures were performed in accordance with the 1964 Declaration of Helsinki and its later amendments or comparable ethical standards.

## Supplementary Information

Supplementary Information.

## Data Availability

The datasets generated and/or analysed during the current study are available in the NCBI GEO database (https://www.ncbi.nlm.nih.gov/geo/). The accession number for the training set data is GSE226289, and the accession numbers for the test set data are GSE157284 and GSE135565.
