# Author Correction: Luminal androgen receptor subtype and tumor-infiltrating lymphocytes groups based on triple-negative breast cancer molecular subclassification

**DOI:** 10.1038/s41598-025-92281-5

**Published:** 2025-03-10

**Authors:** Miseon Lee, Tae-Kyung Yoo, Byung Joo Chae, Ahwon Lee, Yoon Jin Cha, Jieun Lee, Sung Gwe Ahn, Jun Kang

**Affiliations:** 1 Department of Hospital Pathology, Seoul St. Mary’s Hospital, College of Medicine, The Catholic University of Korea, Seoul, Republic of Korea; 2 Division of Breast Surgery, Department of Surgery, Asan Medical Center, University of Ulsan College of Medicine, Seoul, Republic of Korea; 3 Department of Surgery, Samsung Medical Center, Sungkyunkwan University School of Medicine, Seoul, Republic of Korea; 4 Cancer Research Institute, The Catholic University of Korea, Seoul, Republic of Korea; 5 Department of Pathology, Gangnam Severance Hospital, Yonsei University College of Medicine, Seoul, Republic of Korea; 6 Institute of Breast Cancer Precision Medicine, Yonsei University College of Medicine, Seoul, Republic of Korea; 7 Division of Medical Oncology, Department of Internal Medicine, Seoul St. Mary’s Hospital, The Catholic University of Korea, Seoul, Republic of Korea; 8 Department of Surgery, Gangnam Severance Hospital, Yonsei University College of Medicine, Seoul, Republic of Korea; 9 Institute for Breast Cancer Precision Medicine, Yonsei University College of Medicine, Seoul, Republic of Korea

Correction to: *Scientific Reports* 10.1038/s41598-024-61640-z, published online 17 May 2024

The original version of this Article contained an error in the Author information section.

“These authors contributed equally: Miseon Lee, Tae-Kyung Yoo, Sung Gwe Ahn and Jun Kang.”

now reads:

“These authors contributed equally: Miseon Lee and Tae-Kyung Yoo.

These authors jointly supervised this work: Sung Gwe Ahn and Jun Kang.”

The original Article has been corrected.

