# Supplementary material for: Luminal androgen receptor subtype and tumor-infiltrating lymphocytes groups based on triple-negative breast cancer molecular subclassification
Source: Sci Rep. 2024 May 17;14:11278. doi: 10.1038/s41598-024-61640-z (PMC11101432; doi:10.1038/s41598-024-61640-z)
Supplement: Supplementary file 1 — Supplementary Information. [file 41598_2024_61640_MOESM1_ESM.docx]

**Supplementary Table S1**. **Clinical characteristics and TNBC molecular subclassification of the training set and test set**

|  | **Training set**  **(N=142)** | **Test set**  **(N=175)** | ***p*-value** |
| --- | --- | --- | --- |
| Age, years (mean (SD)) | 54.2 (13.0) | 51.7 (21.9) | 0.233 |
| Histologic diagnosis, N (%) |  |  | 0.044 |
| Invasive breast carcinoma, NST | 106 (74.6) | 137 (78.3) |  |
| Carcinoma with medullary features | 11 (7.7) | 16 (9.1) |  |
| Metaplastic carcinoma | 17 (12.0) | 6 (3.4) |  |
| Carcinoma with apocrine differentiation | 2 (1.4) | 7 (4.0) |  |
| Invasive lobular carcinoma | 3 (2.1) | 1 (0.6) |  |
| Salivary gland-like carcinoma | 1 (0.7) | 3 (1.7) |  |
| Other | 2 (1.4) | 5 (2.9) |  |
| Histologic grade, N (%) |  |  | 0.001 |
| 1 | 0 (0.0) | 11 (6.5) |  |
| 2 | 20 (14.2) | 38 (22.5) |  |
| 3 | 121 (85.8) | 120 (71.0) |  |
| Tumor size, cm (mean (SD)) | 2.5 (1.2) | 2.5 (1.5) | 0.761 |
| AJCC pathologic stage, N (%) |  |  | 0.150 |
| 1 | 45 (31.7) | 69 (39.4) |  |
| 2 | 86 (60.6) | 88 (50.3) |  |
| 3 | 11 (7.7) | 15 (8.6) |  |
| 4 | 0 (0.0) | 3 (1.7) |  |
| TNBC molecular subclassification, N (%) |  |  | 0.700 |
| LAR | 21 (14.8) | 24 (13.7) |  |
| IM | 31 (21.8) | 31 (17.7) |  |
| BL1 | 25 (17.6) | 37 (21.1) |  |
| M | 38 (26.8) | 42 (24.0) |  |
| UNC | 27 (19.0) | 41 (23.4) |  |

TNBC, triple-negative breast cancer; SD, standard deviation; NST, no special type; AJCC, The American Joint Committee on Cancer; LAR, Luminal androgen receptor; IM, Immunomodulatory; BL1, basal-like 1; M, Mesenchymal; UNC, Unclassified


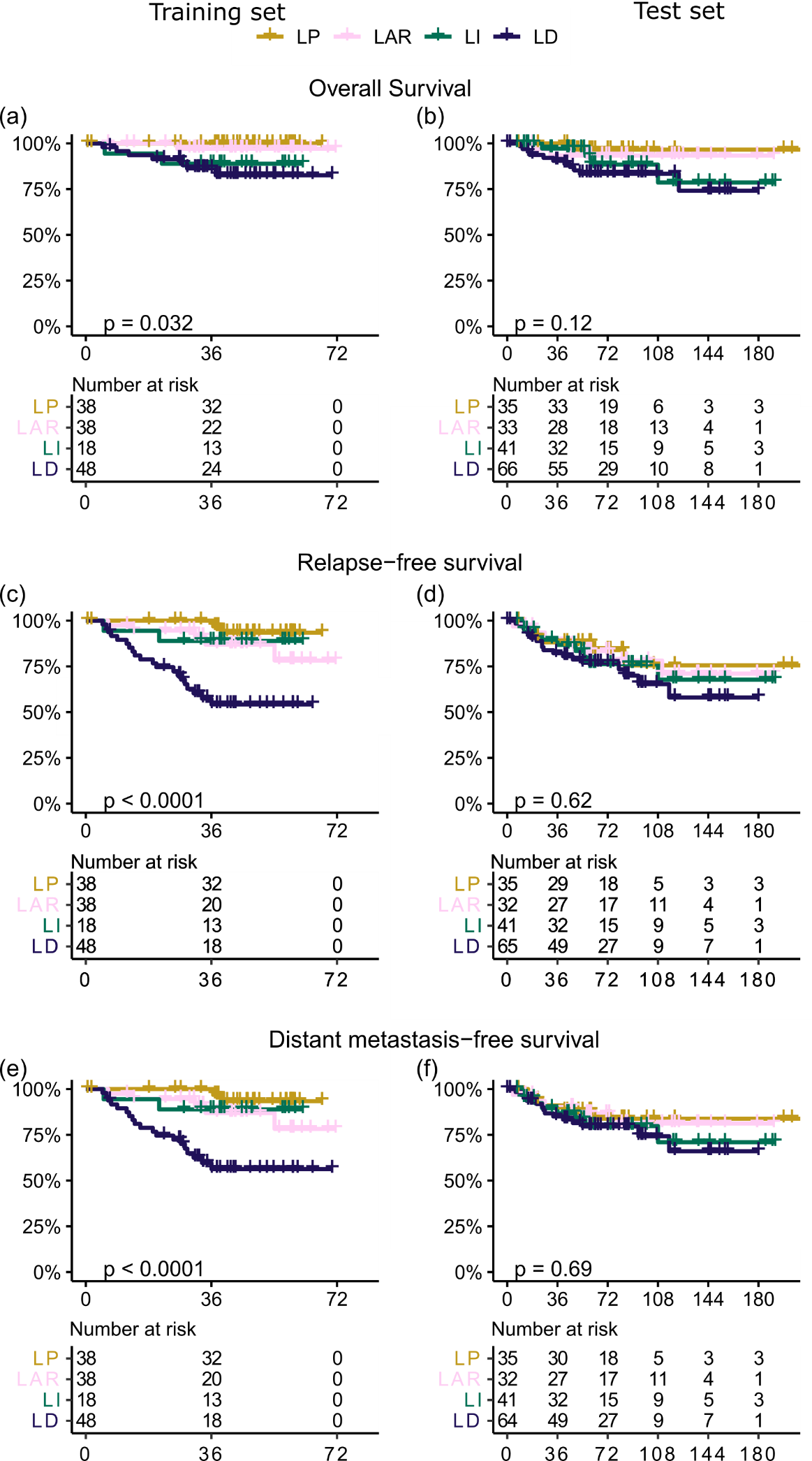


**Supplemental figure 1.** **Survival analysis in each training and test set.** Kaplan-Meier curve for overall survival (a, b), relapse-free survival (c, d), and distant metastasis-free survival (e, f) according to the TNBC subtype classification. The left side is for the training set (a, c, and e) and the right side is for the test set (b, d, and f). LAR, Luminal androgen receptor; LP, Lymphocyte-predominant; LI, Lymphocyte-intermediate; LD, Lymphocyte-depleted.
